# Online Illicit Drug Distribution in the Thai Language on X: Exploratory Qualitative Content Analysis

**DOI:** 10.2196/71703

**Published:** 2025-09-02

**Authors:** Francois Rene Lamy, Seung Chun Paek, Natthani Meemon

**Affiliations:** 1 Health Solutions Research Unit Faculty of Social Sciences and Humanities Mahidol University Bangkok Thailand; 2 Department of Society and Health Faculty of Social Sciences and Humanities Mahidol University Bangkok Thailand

**Keywords:** X, digital drug trade, Thailand, qualitative content analysis, social media data

## Abstract

**Background:**

By increasing exposure to drug-related advertisements, the illicit digital drug trade promotes drug normalization and eases access to substances, increasing the likelihood of initiation. Social media platforms play an increasingly important role in facilitating the online substance trade by leveraging encrypted communications and user-friendly interfaces to advertise a large variety of readily available substances. Despite its growing importance, there is a paucity of research conducted in Thailand that aims to determine the types of substances, marketing strategies, and public health risks linked to drugs advertised on social media.

**Objective:**

This study aimed to inductively explore the content of tweets on the social media platform X (formerly known as Twitter) advertising drugs in the Thai language.

**Methods:**

Tweets advertising psychoactive substances in the Thai language were collected manually between April and July 2024. A qualitative content analysis was performed on the collected tweets. Tweets were coded based on 5 themes: types of substances advertised, marketing strategies, delivery methods, number of substances per tweet, and location references. The intercoder reliability for each theme was assessed using Krippendorff α, achieving substantial agreement across most codes.

**Results:**

A total of 3832 tweets advertising drugs were collected and analyzed. Most tweets (2424/3832, 63.26%) mentioned 5 or more substances, with depressants such as opioids (2807/3832, 73.25%), antihistamines (2394/3832, 62.47%), and benzodiazepines (2009/3832, 52.42%) being the most frequently advertised. Common marketing techniques included direct contact information (2848/3832, 74.32%) and fast delivery (1216/3832, 31.73%). Delivery methods primarily involved courier services but generally offered multiple options. Tweets that mentioned at least 1 sex-performance enhancer were frequently (422/543, 77.7%) advertised in combination with benzodiazepine.

**Conclusions:**

The results of this study suggest the presence of a large number of substances advertised for sale on the X platform in the Thai language. This digital form of drug trading is facilitated by possible direct messaging and the large number of courier services existing in Thailand. Our findings call for the development of real-time monitoring systems that harness drug-related data from social media to inform public health practitioners about emerging substances and trends and address the challenges posed by the digital drug trade.

## Introduction

### Background

The rapid and constant development of digital technologies has gradually facilitated the transactions of illicit substances, from the first transaction in 1972 between university students using their institutions’ Arpanet accounts (email predecessor) [1] to current social media–based purchases. In the early 2000s, Clearnet or Surface websites sold semi-illicit substances, such as pharmaceutical drugs without a prescription and novel substances listed as “plant food,” “research chemicals,” or “spice” and advertised as “not for human consumption” [2-4]. A decade later, in the early 2010s, most of the online drug distribution transitioned to darknet markets, also called “cryptomarkets.” Cryptomarkets are online marketplaces located within the “Darknet” that rely on advanced encryption techniques and cryptocurrencies (eg, Bitcoin and Monero) to provide anonymity to merchants and buyers [5].

More recently, results from several international studies suggest that psychoactive substances are now increasingly advertised on the “clearnet” (ie, the portion of the internet that can be accessed through classic web browsers). Since the end of the 2010s, messaging services offering advanced encryption and real-time group messaging [6-10], as well as social media applications allowing interactions and user-generated content sharing, have started taking an important role in digital drug trading [11-15]. It is important to note that in the Thai context, social media platforms tend to be used as advertising platforms. Substances are advertised using images and specific keywords that can be searched or directly seen in the user’s feed, while “messaging services” (eg, Line) are ultimately used as communication means, allowing encrypted direct messaging between the seller and potential buyer [16]. More precisely, drug dealers have started using Discord servers [13] or messaging services such as Signal and WhatsApp to directly reach out to their customers or repetitively advertise their products on popular social media platforms, such as X (formerly known as Twitter), Telegram, TikTok, and Instagram [16-21]. The ease of access to online markets combined with a friendly user interface and integrated anonymity tools has transformed “classical” drug buying experiences by limiting the chance of arrest, reducing violence, and offering convenient options for drug purchase [22,23].

In addition, the illicit distribution of drugs through online media has rapidly expanded during the COVID-19 pandemic. The pandemic has accelerated the development and use of e-commerce and delivery or courier systems (eg, Grab, Lineman, etc), offering new alleys for drug distribution. The combination of quarantine and increased accessibility of drugs online has led numerous drug users to purchase their drug of choice through the internet. Results from the Global Drug Survey indicate that the percentage of respondents who have used any online platforms to purchase drugs went from 4.7% in 2014 to 15% in 2020 [24]. Meanwhile, the number of online pharmacies and websites advertising pharmaceutical products has also grown rapidly in the past decade, increasing the likelihood of potential misuse of pharmaceutical products with psychoactive effects [25]. Overall, online media facilitates the distribution of a wider variety of drugs by making them readily available and easier to access by having the products come to the buyer rather than the user going to the dealer [7].

Although the number of studies investigating the digital drug trade has drastically grown in recent years, these studies were generally conducted in Western settings, while a limited number of such studies focused on Asian countries. For example, in Thailand, only 2 studies have investigated the illicit distribution of drugs through online media. Thaikla et al [26] collected Facebook posts related to kratom and cannabis in the Thai language from 2015 to 2016. Their results suggested that most Facebook users had a positive attitude toward both cannabis and kratom, that they were advertised online, and that posts did not contain warnings concerning potential negative health effects linked to cannabis or kratom [26]. Pinyopornpanish et al [27] studied the availability of sedatives and analgesics on websites and Facebook. Their results indicate that these drugs were advertised on a large number of Thai websites and 14 Facebook pages. None of these pages or websites contained information about potential harms linked to sedative or analgesic use, and several advertised these substances as potential “date-rape drugs” [28]. These 2 investigations were pioneers in Thailand and provided early snapshots of how online distribution has penetrated Thai social media. However, these 2 studies did not aim to characterize the type and volume of substances advertised on Thai social media. These studies have not been followed by more extensive research aiming to deepen and broaden our understanding of this phenomenon.

### This Study

Despite the growing volume of psychoactive substances advertised and purchased online, there is a dearth of research on the types and characteristics of substances available online in Thailand, as well as the semantic content of such advertisements. This study aimed to provide initial insights into the content of tweets illicitly advertising psychoactive substances on X in the Thai language. X is frequently used for substance use surveillance as it contains unedited casual disclosures of “day-to-day” drug encounters as well as illegal or deviant behaviors [17,29-37]. Moreover, X users are mostly young and young adults (Insider Intelligence [38]), which constitute the main age groups consuming psychoactive substances in Thailand [39]. Indeed, X has grown in popularity in recent years, with over 14.7 million active users in early 2024, representing 20.4% of the Thai population [40].

## Methods

### Data Collection

Tweets illicitly advertising psychoactive substances on X in the Thai language were collected from April 1, 2024, to July 31, 2024. As collecting data from X for free is now limited to a maximum of 1500 tweets per month, and that X robot [41] explicitly forbids the use of any automated “bot” to collect most data from its website, a research assistant was tasked to manually collect tweets based on Thai drug-related keywords weekly. These keywords were selected based on a snowballing technique consisting of 3 steps: first, several tweets were queried based on a few initial drug-related Thai search terms (eg, 
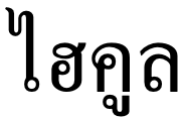
 [HiCool], “
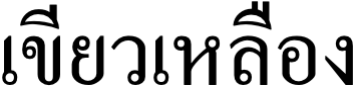
” [green-yellow], “
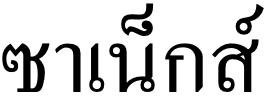
” [Xanax], “
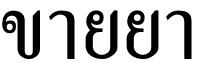
” [sell drugs], and “
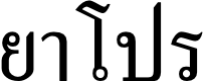
” [Promethazine] [42,43]); second, keywords and hashtags used to describe substances in the collected tweets advertising drugs were identified, and; third, accuracy of new potential keywords was checked by a Thai native speaker (NM) and added to the list of search terms if deemed accurate. This process was repeated until no new drug-related keywords were identified. A total of 88 keywords were identified through this process (Multimedia Appendix 1). This inductive process does not allow for the list of psychoactive substances used as keywords to be exhaustive, but it does allow for the search terms to be contextualized; therefore, they are more likely to be accurate in the X environment. Moreover, manual search does not allow for filtering of tweets according to language; therefore, the list of search terms aimed not to include terms in English to avoid noise.

Tweets were collected based on several inclusion and exclusion criteria. Inclusion criteria were as follows: tweets that (1) were publicly available, (2) contained mention of at least 1 psychoactive substance illicitly advertised, and (3) clearly indicated an intention to sell a product (eg, mention of gram per Thai Bhat, delivery methods, or sale-related terms) were collected. Tweets advertising solely cannabis were excluded from this study as both the medical and recreational use of cannabis were legal in Thailand at the time of data collection [44-46]. In addition, tweets describing user experiences, prevention messages, or news media articles were also excluded.

The research assistant connected to X weekly to retrieve tweets of interest based on the aforementioned inclusion and exclusion criteria, manually copying the text and date and time of each identified tweet in a single CSV file. Duplicated tweets were discarded based on the date and time and text of collected tweets at the end of the data collection period. Tweets advertising solely e-cigarettes (or tobacco cigarettes) were also removed from this analysis due to their large number requiring a separate study dedicated to the topic. It is also important to note that the manual data collection was not limited to tweets posted during the data collection period but also contains tweets before May 2024.

### Data Analysis

Texts manually extracted from collected tweets were then analyzed using a qualitative content analysis approach. This type of analysis is conducted in three overlapping stages: (1) development of a coding scheme by a team of coders, who have read and reread an initial sample of tweets; (2) consistent application of codes to the entire body of the tweets, ensuring that meanings are not lost; and (3) an interpretation and analysis phase that aims to establish a pattern for the whole by relating the codes to one another [47]. The “coding scheme” was developed by coding a subset of 500 random tweets from the total dataset of 3832 collected tweets. This subset was used to explore meanings, discuss discrepancies, and develop and refine the coding rules. The inductive approach in qualitative coding, which is also referred to as “open coding” [48], moves from the specific to the general (bottom-up approach) and allows examination of phenomena within their own context rather than from a predetermined conceptual basis. Intercoder reliability was tested for each category using Krippendorff α [49]. Krippendorff α of 0.667 to 0.80 indicates moderate agreement, and greater than 0.80 indicates substantial agreement. On the basis of the preliminary discussion among coders, the coding scheme included the codes shown in Textbox 1.

**Coding schemes and codes.**

**Number of substances advertised**

- Collected tweets tend to advertise several substances in the same post. The number of substances was categorized as “1,” “2 to 4,” and “≥5.”

**Marketing strategies**

- Five selling techniques were detected during the initial discussion among coders.
  Discount: sellers offered a discount for higher purchase volumes.
  Credibility: sellers specified that the ordered products will be delivered.
  Fast delivery: sellers described 1 or several methods for the products to be delivered in a short period.
  Direct contact: sellers provide their Line ID or telephone number to be directly contacted by potential buyers.
  Quality: sellers describe their products as high quality, potent, and pure.

**Location**

- During their initial exploration of 500 random tweets, the coders noticed that some tweet texts contained a mention or specification of a province (eg, Chiang Mai and Nonthaburi) or a town or a location (eg, Pattaya and Sukhumvit Soi 4), most likely to provide information regarding the area or town where the dealer can meet or send the purchased products by courier.

**Types of drugs**

- The substances advertised in the 500 initial tweets were categorized into 11 classes
  Amphetamine-type substances: “Yaba” (methamphetamine in a pill form) and “YaIce” (methamphetamine in crystal form)
  Opioids: both illicit (eg, opium or heroin) or medical (eg, Tramadol or Neocodion)
  Benzodiazepine: by chemical name (eg, alprazolam and flunitrazepam), brand name (eg, Xanax), and slang terms (eg, zolam)
  Antihistamine: promethazine and Procodyl
  Hallucinogens: magic mushrooms and lysergic acid diethylamide
  Cannabis (in addition to other substances)
  Sex-performance substances: Viagra, Kamagra, gamma-hydroxybutyrate, and gamma-butyrolactone
  Cocaine
  Ketamine
  Ecstasy or 3-methoxy-4,5-methylenedioxyamphetamine
  Others

**Methods of delivery**

- Three main types of delivery methods were found in the initial tweets
  Courier: sellers indicate that the purchased products can be delivered at the doorstep of the buyer using a courier service, such as Lineman or Grab
  Postal: seller would post the substances bought using one of the tracking postal services available in Thailand (ie, Kerry Express or Express Mail Service)
  Face-to-face: the seller proposed to meet the buyer if located in the same vicinity
- Because a large part of the initial 500 tweets offered the 3 aforementioned options cumulatively, a “multiple” code was created to complete this theme.

### Ethical Considerations

The Mahidol University Social Sciences Institutional Review Board approved the research (2024/032.2202).

## Results

### Data Collection

Over the 4 months of the data collection period, 3832 unique tweets were collected. These 3832 tweets were divided among 3 Thai native coders for content analysis.

### Intercoder Reliability Results

The results of the intercoder reliability assessment are displayed in Table 1.

To the exception of the marketing strategy “quality” code and to a lesser extent “antihistamine” type of substance, the 3 coders achieved a Krippendorff α score greater than 0.8 across all other codes, indicating substantial agreements.

**Table 1:** Intercoder reliability assessment results.

Themes			Average pairwise agreement (%)		Krippendorff α
Number of drugs advertised			90		0.84
Location			96		0.83
**Marketing strategies**					
	Discount	97.1		0.89	
	Credibility	95		0.82	
	Fast delivery	97.1		0.89	
	Direct contact	97.8		0.95	
	Quality	93.4		0.65	
	Delivery method	97.2		0.93	
**Types of substances**					
	Amphetamine-type substances	98.3		0.88	
	Opioid	94.2		0.87	
	Benzodiazepine	94.1		0.87	
	Antihistamine	89.2		0.74	
	Hallucinogens	99.9		0.88	
	Cannabis	98.4		0.80	
	Sex-performance substances	97.3		0.81	
	Cocaine	99.8		0.88	
	Ketamine	99.4		0.80	
	MDMA^a^-type substance	99.3		0.94	

^a^MDMA: 3-methoxy-4,5-methylenedioxyamphetamine.

### Qualitative Content Analysis Results

Multimedia Appendix 2 displays the overall results of the qualitative content analysis based on the codes and themes that emerged during initial exploration.

Most collected tweets (2424/3832, 63.3%) advertised 5 or more substances (Table 2). This can denote that either online sellers are generally advertising a wide range of substances or that they intentionally added drug-related hashtags or keywords to increase the range of X users that will be exposed to their advertisements. Specific locations or provinces were advertised in slightly more than a third (1322/3832, 34.5%) of collected tweets. This percentage can be explained by the different methods of delivery used by X sellers (described subsequently).

Most marketing strategies were found in less than half of the collected tweets, with the exception of “direct contact.” In total, 74.32% (2848/3832) of the collected tweets contained a Line ID or a telephone number to enable direct messaging or communication between potential buyers and sellers.

Nearly half (1895/3832, 49.45%) of collected tweets did not specify any delivery method. Most of the tweets (1596/3832, 41.57%) that contained a delivery method offered several options for purchased products to be delivered. If only 1 method was proposed, “postal” and “face-to-face” were the least common, with 0.84% (32/3832) and 1.33% (51/3832) of collected tweets, respectively.

**Table 2:** Most common substances advertised in combination.

Most frequent drug in combinations	Opioids (2807/3832, 73.25%), n/N (%)	Antihistamines (2394/3832, 62.47%), n/N (%)	Benzodiazepines (2009/3832, 52.43%), n/N (%)	Amphetamine-type substances (756/3832, 19.73%), n/N (%)	Ketamine (546/3832, 14.25%), n/N (%)	Sex-performance substances (543/3832, 14.17%), n/N (%)	MDMA^a^-type substances (513/3832, 13.39%), n/N (%)	Cannabis (426/3832, 11.12%), n/N (%)
Opioids	—^b^	2236/2394 (93.4)	1535/2009 (76.41)	427/756 (56.5)	397/546 (72.7)	244/546 (44.9)	361/513 (70.4)	393/426 (92.3)
Antihistamines	2236/2807 (79.69)	—	1525/2009 (75.91)	41/756 (5.4)	140/546 (25.6)	251/546 (46.2)	76/513 (14.8)	371/426 (87.1)
Benzodiazepines	1535/2807 (54.68)	1525/2394 (63.7)	—	72/756 (9.5)	184/546 (33.7)	422/546 (77.7)	108/513 (21.1)	282/426 (66.2)
Amphetamine-type substances	427/2807 (15.22)	41/2394 (1.71)	72/2009 (3.58)	—	371/546 (67.9)	3/546 (0.6)	430/513 (83.8)	16/426 (3.8)
Ketamine	396/2807 (14.11)	139/2394 (5.80)	185/2009 (9.21)	371/756 (49.1)	—	92/546 (16.9)	420/513 (81.9)	20/426 (4.7)
Sex-performance substances	244/2807 (8.69)	251/2394 (10.48)	422/2009 (21.00)	3/756 (0.4)	92/546 (16.8)	—	11/513 (2.1)	4/426 (0.9)
MDMA-type substances	362/2807 (12.89)	77/2394 (3.21)	108/2009 (5.37)	430/756 (56.9)	420/546 (76.9)	11/546 (2)	—	20/426 (4.7)
Cannabis	393/2807 (14.00)	371/2394 (15.49)	281/2009 (13.99)	16/756 (2.1)	20/546 (3.7)	4/546 (0.7)	20/513 (3.9)	—

^a^MDMA: 3-methoxy-4,5-methylenedioxyamphetamine.

^b^Not applicable.

Concerning the types of substance advertised, depressant-type drugs, opioids (2807/3832, 73.25%), antihistamines (2394/3832, 62.47%), and benzodiazepines (2009/3832, 52.42%) were the 3 most frequently advertised substances. Stimulant-type drugs were less frequently advertised with amphetamine- and MDMA (3-methoxy-4,5-methylenedioxyamphetamine)-type substances present in 19.72% (756/3832) and 13.39% (513/3832) of collected tweets, respectively. Ketamine and sex-performance substances were advertised in 14.24% (546/3832) and 14.17% (543/3832) of collected tweets, respectively. Cannabis products (in addition to other substances) were advertised in 11.12% (426/3832) of tweets. Rarely advertised substances were cocaine (59/3832, 1.54%) and hallucinogens (44/3832, 1.15%). Notably, 
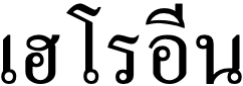
 (heroin) was mentioned in only 4.59% (129/2807) of tweets that advertised opioids (opium, synthetic heroin, and fentanyl were not mentioned), indicating that most of the advertised opioid substances were diverted medications.

### Most Common Substances Advertised in Combination

The most common classes of substances advertised in combinations with each other were determined (Table 2). Cocaine and hallucinogens were removed from this calculation due to their infrequent mention in collected tweets.

Depressant substances (ie, opioids, benzodiazepines, and antihistamines) were frequently advertised in combination. Cannabis was also frequently advertised with these substances. Stimulant-type substances (ie, amphetamine- and MDMA-type substances) were frequently advertised together as well as with opioids and ketamine. Sex-performance enhancers were frequently found in combination with depressant substances, especially benzodiazepines: 77.7% (422/543) of advertisements that contained at least a mention of a sex-performance enhancer also contained at least 1 mention of a benzodiazepine).

## Discussion

### Principal Findings

This exploratory study aimed to provide initial insights regarding the content of tweets illegally advertising psychoactive substances in the Thai language. The results of the qualitative content analysis suggest that a large variety of drugs are available online and that most of the substances illegally advertised on X are from the depressant class of drugs. Sellers also engage in different marketing techniques to draw the attention of potential buyers and facilitate purchases through private communication and various delivery methods. Our results indicate that most of the collected tweets illegally advertising psychoactive substances cover multiple drugs at once, with a large proportion marketing 5 or more substances. To the best of our knowledge, this is one of the first qualitative content analyses of tweets that illegally advertise drugs online and, more specifically, in the Thai context.

As mentioned previously, most of these tweets were from the depressant class of drugs that were frequently advertised together. The polyuse of depressant-type substances, especially in cases where opioids are involved, could have severe harmful consequences. Although benzodiazepines are generally combined with opioids to enhance their effects [50,51], their combination has been linked to acute respiratory depression [52] and could potentially cause unintentional overdose deaths [53]. Similar to benzodiazepines, antihistamine medications, originally used to treat allergies, can exacerbate opioid induced sedation [54], potentially heightening chances of overdose [55]. It is important to note that an antihistamine mixed with opioids and a soft drink, a cocktail named “YaPro” in Thailand, is prevalent among young Thai users and has been linked to transitioning to heroin among young Thai males [42]. In addition, antihistamines advertised online could be counterfeit, potentially leading to additional adverse consequences. At least 2 illegal laboratories producing counterfeit antihistamines were raided in Thailand a few months before this manuscript was written [56,57]. The recreational misuse of medications is not a new phenomenon in Thailand. Chittrakarn and Assanangkornchai [58] investigated the misuse of therapeutic drugs, indicating the important role played by online shops in facilitating the purchase of such substances. However, the impact of social media, and more specifically, how social media use influences the online purchase of drugs, remains unknown in Thailand. This calls for additional research on the topic, whose results can help develop education and prevention messages limiting the potential harmful consequences linked to depressant polyuse.

Although depressant-type drugs were more frequently advertised compared to stimulant-type drugs, methamphetamine (in both pill and crystal forms) remains the most commonly used illicit substance in Thailand [59]. This can be at least partially explained by the fact that this study only collected tweets from X. Future research should collect data from several social media and messaging apps to establish if specific substances are more or less prevalent and can be sourced more or less easily on specific platforms. It also calls for understanding to what extent Thai people source methamphetamine through an online or offline mode.

Another important finding from this research is that sex-performance enhancer–type drugs were frequently found to be advertised in combination with depressants, especially substances from the benzodiazepine class. Benzodiazepines can be misused to induce sedation and lethargy in a person without their knowledge. Flunitrazepam or Rohypnol, called the “Blue Tongue” drug in Thailand, has been detected from 2022 to 2023 [60,61] and has been linked with several hospitalizations. There is a paucity of research in Thailand on the prevalence of benzodiazepine use in the general population, and among the youth in particular, as well as the number of emergency visits linked to the unintentional consumption of benzodiazepines.

While it is known that social media drug dealing is “between cryptomarkets and street-based drug markets” [62], involving online advertisements finalized by a physical encounter “face-to-face” (if not combined with other modes of delivery, such as “courier” and “postal”) was one of the least frequent mode of delivery proposed in collected tweets that contain mentions of any delivery method. This could be explained by the ubiquitous presence of courier-based services in Thailand (eg, Grab and Lineman) and by the ease of using such services. In contrast with Western settings, this specificity warrants further investigations to assess the volume of courier-based drug delivery through a consumer-based survey. In addition, advertisements collected during this study tend to encompass several marketing techniques, such as bulk orders, discounting, or “credit score.” These strategies, frequently used in e-commerce, suggest the existence of competition in the Thai illicit digital drug market as well as the presence of scammers. Understanding the perceived benefits and negative experiences encountered by Thai people who order drugs online would help identify potential risks and related harms inherent to illicit digital drug purchases. This information can then be used to design evidence-based prevention and harm minimization messages that can be disseminated on social media of interest.

Furthermore, sellers appear to frequently provide a telephone number or a messaging app ID (eg, Line direct messaging app) to facilitate communication with potential buyers, which echoes findings from research conducted in other countries [62]. It needs to be specified that Line app messaging is fully encrypted, guaranteeing that communication between sellers and buyers remains anonymous and Line application programming interface does not offer the option of crawling user-generated content. Digital ethnography studies could investigate Line groups dedicated to drug use in the Thai language to gain additional information regarding the digital drug trade in Thailand.

Overall, monitoring online advertisements of drugs sold illicitly can provide early insights on both existing and emerging trends as well as novel substances available on the Thai drug market. Additional efforts should be dedicated to creating and deploying a monitoring system able to collect and analyze not only drug-related advertisements in a timely manner, but also user-generated content linked to substance use in order to better inform practitioners as well as the general population. In its 2023 report, the International Narcotic Control Board recommended addressing the illicit digital drug trade by combining law enforcement actions and public health harm reduction and prevention campaigns to raise awareness and educate the general population [63]. In the same report, the International Narcotic Control Board also proposed that governments work in partnership with tech companies to detect and timely remove illegal content, such as drug advertisements. In Thailand, efforts have mostly focused on arresting online dealers; for example, the Thai Royal Police cooperated with postal delivery agencies, such as the Thailand Post and Kerry Express, to arrest several dealers [64]. However, a recent report from the Thai International Technology Transfer Center underscored that drugs were still advertised on social media (primarily X and Facebook), targeting young adults through 24-hour delivery, cash-on-delivery, and promotional assurances [65]. Dealer arrests or website and market closures tend to redirect the digital drug trade to other vendors or new websites [66]. The results from the Thailand International Technology Transfer Center investigation and from this study suggest that the illicit digital drug trade remains persistent in Thailand. Concerning harm minimization and prevention efforts, Thai nongovernmental organizations (especially the O-Zone Foundation or the Thai Drug Users’ Network) have successfully reduced harms linked to injection drug use; however, to the best of our knowledge, there is no online service dedicated to inform and educate social media users about the potential threats involved in online drug purchasing. Such an online information platform could help reduce the burden linked to the illicit digital drug trade.

Despite the difficulties inherent in collecting data from social media for free, future similar studies should aim to obtain a larger dataset based on additional keywords and for an extended period. Future research could use the keyword snowballing method used in this study and combine slang terms with official drug-related terms in order to maximize the number of relevant context-based keywords for data queries. In addition, images from posts illicitly advertising substances could be collected and analyzed to further enhance the findings (eg, collect brand names, verify the type of substance advertised, etc). The question about how social media use (eg, time spent online and platforms used) affects this form of purchase should also be addressed by conducting an online survey among people who buy drugs online. Finally, future national household surveys should incorporate questions that pertain to online drug buying activities to gain insights regarding the prevalence of illicit online drug purchases in Thailand.

### Limitations

This study is not without limitations. Most of them are inherent in the field of social media data analysis. First, data collection was not exhaustive. As of July 11, 2023, X has decided to revert the access of “academic” accounts to “free” accounts, which limited the number of tweets that can be collected from 10 million to 1500 per month. This reversal prevents the ability to automatically collect a large volume of tweets through their application programming interface for free; hence, our data collection might not have captured the totality of tweets advertising illicit drugs. While the manual collection used for this exploratory study yields enough data, it is a tedious and inefficient method that can only produce (from our understanding) partial results. Second, data collection was limited to tweets written in the Thai language, which does not guarantee that all collected tweets were posted from within the Kingdom of Thailand. Third, there is no certainty that the sellers illegally advertising the substances found in the collected tweets were really in possession of these drugs. Some vendors were most likely scammers, impacting the final results of this exploratory study.

### Conclusions

In conclusion, our analysis demonstrates that a wide variety of substances, particularly depressant-type drugs, are frequently advertised on the Thai X platform. Dealers also appear to leverage courier services and anonymity tools inherent to social media apps to facilitate drug distribution. Importantly, a large number of collected tweets advertised several substances in combination, heightening the risks of health-related harms linked to (intentional or unintentional) polysubstance use. This exploratory study provides preliminary data on understanding the scope of the digital drug trade in Thailand. The results of this study also call for the urgent development of real-time monitoring systems harnessing drug-related data from social media. Such a system can better inform public health practitioners about emerging substances and trends and ultimately help address the challenges posed by the digital drug trade in Thailand.

## Appendix

Multimedia Appendix 1 Search terms used to collect relevant tweets in Thai language with their English translation.

Multimedia Appendix 2 Results of the qualitative content analysis of collected tweets.

## Abbreviations

MDMA: 3-methoxy-4,5-methylenedioxyamphetamine
