# Supplementary material for: Online Illicit Drug Distribution in the Thai Language on X: Exploratory Qualitative Content Analysis
Source: JMIR Infodemiology. 2025 Sep 2;5:e71703. doi: 10.2196/71703 (PMC12441646; doi:10.2196/71703)
Supplement: Multimedia Appendix 1 [file infodemiology_v5i1e71703_app1.docx]

Search terms used to collect relevant tweets in Thai Language with their English translation

| **Drug Category** | **Thai Terms** | **English Translation** | **Note** |
| --- | --- | --- | --- |
| Antihistamine – Slang Terms | ฝาแดง | Red cap | Antihistamine syrup |
| Antihistamine Slang Terms | ยาแก้ไอฝาแดง | Red cap cough medicine | Antihistamine syrup |
| Antihistamine -Promethazine | โปร | Pro | Slang for Promethazine |
| Antihistamine -Promethazine | โปเมทาซีน | Promethazine | A brand name of Antihistamine |
| Antihistamine -Promethazine | โปรเมทาซีน | Promethazine | A brand name of Antihistamine |
| Antihistamine - Cetirizine | สตารเทค | Startec | A brand name of cetirizine hydrocholoride |
| Antihistamine - Cetirizine | สตาร์เทค | Startec | A brand name of cetirizine hydrocholoride |
| Antihistamine | ฟาเทค | Fatec | A brand name of Antihistamine |
| Antihistamine | กินโปร | Eat pro | Slang for Promethazine |
| Benzodiazepine - Alprazolam | โซแลม | Zolam | Slang for Alprazolam |
| Benzodiazepine - Alprazolam | โซแล่ม | Zolam | Slang for Alprazolam |
| Benzodiazepine - Alprazolam | ออแล่ม | Olam | Slang for alprazolam |
| Benzodiazepine - Diazepam | d10 | D10 | Slang for diazepam pills |
| Benzodiazepine - Diazepam | มไดอาซีแพม | Diazepam | N/A |
| Benzodiazepine - Diazepam | โซแพม10 | Zopam10 | Slang for Diazepam |
| Benzodiazepine - General Terms | ยาตบ | Slap drugs | Benzodiazepines used to cause unconsciousness |
| Benzodiazepine - Rohypnol | โรฮิบนอล542 | Rohypnol542 | Date rape drug |
| Benzodiazepine - General Terms | ยาลิ้นฟ้า | Blue tongue drugs | Rohypnol |
| Benzodiazepine - Lorazepam | ลอร่าซีแพม | Lorazepam | N/A |
| Drug - General terms | เม็ดเมา | Drug | N/A |
| Drug - General terms | ยาชุด | Drug set | N/A |
| Drug - General terms | ยาเมา | Drunk drugs | N/A |
| Drug - General terms | สายเมา | Drunk people | N/A |
| Drug - General terms | ยาแก้ปวด | Pain relievers | N/A |
| Drug - General terms | สายตี้ | Party people | N/A |
| Drug - General terms | ขายยา | Sell drugs | N/A |
| Drug - General terms | ยานอนหลับ | Sleeping pills | N/A |
| Drug - General terms | ยาครูตีไม่เจ็บ | Effective heavy with no pain | Analgesic |
| Drug - General terms | ยาแก้เครียด | Stress relievers | Anxiolytic |
| Drug - General terms | ยาน้ำ | Liquid drugs | N/A |
| Drug - General terms | โรเซ่ | Roche | Sedatives |
| Drug - General terms | ร้านยา | Drugstores | N/A |
| Drug - Others | บี5 | B5 | Pantothenic Acid |
| Drug - Others | บีไฟว์ | B5 | Pantothenic Acid |
| Drug - Others | ยาเม็ดเบ็นซ์เฮ็กซอล | Benzhexol pills | N/A |
| Drug - Others | เคนมผง | K-milk powder | Drug mixture (Ketamine, Heroin, Methamphetamine, Benzodiazepines) |
| Drug - Slang terms for quantity | ตัวใหญ่ | Big one | Bulk transaction or Package of drugs |
| Drug - Slang terms for quantity | งานก้อน | Block work | Bulk transaction or Package of drugs |
| Drug - Slang terms for quantity | แคปชั่ | Capsule | N/A |
| Drug - Slang terms for quantity | แผง | Pack | N/A |
| Drug - Slang terms for quantity | แท่ง | Bar | N/A |
| Ecstasy | เม็ดม่วง | Purple pill | Ecstasy tablet / MDMA pill |
| GHB | ยาจี | G-drugs | GHB: Gamma-Hydroxybutyrate |
| GHB | สารจีเอชบี | GHB substances | N/A |
| Ketamine | เคตามีน | Ketamine | N/A |
| Ketamine | คีตามีน | Ketamine | N/A |
| Ketamine | ยาเค | Ketamine | N/A |
| Ketamine | เคตามี | Ketamine | N/A |
| Methamphetamine | ไฮ | Hi | Methamphetamine |
| Methamphetamine | hicool | Hicool | Thai slang for methamphetamine crystal |
| Methamphetamine | ไฮคลู | Hi-cool | Methamphetamine crystal |
| Methamphetamine | hifun | Hifun | Thai slang for methamphetamine crystal with a sexual connotation |
| Methamphetamine | ไฮเงี่ยน | Hi-horny | Thai slang for methamphetamine crystal with a sexual connotation |
| Methamphetamine | ไฮดีด | Hi-hyped | Methamphetamine |
| Methamphetamine | น้ําแข็ง | Ice | Methamphetamine crystal |
| Methamphetamine | ยาไอซ์ | Ice drugs | Methamphetamine crystal |
| Methamphetamine | ยาบ้า | Methamphetamine | N/A |
| Methamphetamine | ขายยาไอซ์ | Sell ice drugs | N/A |
| Methamphetamine | ขายยาบ้า | Sell methamphetamine drugs | N/A |
| Methamphetamine | เซ่ไทย | Sythai | Methamphetamine |
| Methamphetamine | ซีซ่า | C-Ca, Sisa | Methamphetamine crystal |
| Opioid - Codeine syrup | wocklean | Wocklean | Codeine-based syrup added to soft drink |
| Opioid - Codeine syrup | ลีนแท้ | Real lean | Codeine-based syrup added to soft drink |
| Opioid - Codeine syrup | ลีน | Lean | Codeine-based syrup added to soft drink |
| Opioid - Codeine syrup | ลีนusa | Lean USA | Codeine-based syrup added to soft drink |
| Opioid - Codeine syrup | ลีนนำเข้า | Imported lean | Codeine-based syrup added to soft drink |
| Opioid - Codeine syrup | ลีนนอก | Foreign (imported) lean | Codeine-based syrup added to soft drink |
| Opioid - Codeine syrup | ยาน้ำนอก | Foreign (imported) liquid drugs | Imported codeine syrup |
| Opioid – Codeine | โคดีอีน | Codeine | N/A |
| Opioid - Heroin | เฮโรอีน | Heroin | N/A |
| Opioid - Others | neo-k | Neo-k | Neo-Codion |
| Opioid - Others | ยาแก้ปวดโอพิออย | Opioid pain relievers | N/A |
| Opioid - Others | เฮโรอีนสังเคราะห์ | Synthetic heroin | N/A |
| Opioid - Others | ผงขาว | White powder | Heroin |
| Opioid - Tramadol | ยาแคปซูลสีเขียวเหลือง | Green and yellow capsules | Tramadol is frequently sold in green and yellow capsule |
| Opioid - Tramadol | เขียวเหลื | Green yellow | Drug cocktail containing Tramadol |
| Opioid - Tramadol | เขียวเหลือง | Green yellow | Tramadol |
| Opioid - Tramadol | แผงเขียวเหลือง | Green yellow blister | Tramadol |
| Opioid - Tramadol | แท็กซี่ | Taxi | Taxi can be of yellow and green colors in Thailand, which also are the colors of Tramadol capsules. |
| Opioid - Tramadol | ยาแท็กซี่ | Taxi drugs | Tramadol |
| Opioid - Tramadol | ทรามาดอล | Tramadol | N/A |
| Opioid - Tramadol | ทามาดอล | Tramadol | N/A |
| Opioid - Tramadol | วอลซิดอล | Volcidol | A brand name of Tramadol |
| Opioid - Tramadol | เพนดอล | Panadol | A brand name of Tramadol |
| Pseudoephedrine | คอนดัก | Condect | A brand name of Pseudoephedrine |
| Pseudoephedrine | พีน่า | Pina | A brand name of Pseudoephedrine |
| Sex-enhancer drugs | ยานอนหลับแบบน้ำ | Spanish fly | Aphrodisiac containing cantharidin |
| Sex-enhancer drugs | ยาปลุกเซ็กส์ | Sex stimulants | N/A |

This is a Multimedia Appendix to a full manuscript published in the JMIR Infodemiology journal. For full copyright and citation information see http://dx.doi.org/10.2196/71703.
