# Supplementary material for: Online Illicit Drug Distribution in the Thai Language on X: Exploratory Qualitative Content Analysis
Source: JMIR Infodemiology. 2025 Sep 2;5:e71703. doi: 10.2196/71703 (PMC12441646; doi:10.2196/71703)
Supplement: Multimedia Appendix 2 [file infodemiology_v5i1e71703_app2.docx]

Results of the qualitative content analysis with examples (N=3832).

| Themes | | Tweets, n (%) | Examples in Thai | Examples in English^a^ |
| --- | --- | --- | --- | --- |
| **Number of substances advertised** | | | | |
|  | 1 | 495 (12.9) | #ยานอนหลับยอดนิยม แบบเม็ดโซแลม แท้ เกรดพรีเมี่ยม เก็บเงินปลายทางสายตรง 062-965-xxxx ปอนด์ครับ | #TopSleepingPill Premium-grade genuine zolpidem tablets. Cash on delivery available. Direct line: 062-965-xxxx. Contact: Pond |
|  | 2 to 4 | 913 (23.8) | ซีซ่าขวดล่ะ 65เขียวเหลืองแผงล่ะ55 ซีซ่า1 เขียวเหลือง1=120 ซีซ่า1 เขียวเหลือง2=175#กาญจนบุรี #ราคารับเอง#โปรดีต้องลอง #หาอะไรทักมาได้ครับ | Ciza syrup: 65 THB per bottle. Green-yellow pills: 55 THB per blister pack. Ciza 1 bottle+green-yellow pills 1 pack=120 THB. Ciza 1 bottle+green-yellow pills 2 packs=175 THB. #Kanchanaburi #Self-pickupPrice #GreatDealWorthTrying #MessageMeForAnything |
|  | 5 or more | 2424 (63.3) | #ลีนแท้ #ลีนนอก #ลีนไซรัป #กัญชา #กัญชาog #ทรามาดอล #โปรเมทาซีน #เขียวเหลือง #ยาแก้ไอ #เพนดอล #ทินดอล #เม็ดเมา #promethazine #tramadol #leanUS #fatec #ฟาเทค #ยาน้ำ #ไซรัป #leansyrup #B5 #บี5 #บีไฟว์ #เห็ดเมา #เห็ดวิเศษสอบถามได้คับhttp://line.me/R/xxxxxxxxx… | #AuthenticLean #ImportedLean #LeanSyrup #Cannabis #OGCannabis #Tramadol #Promethazine #GreenYellow #CoughSyrup #Pendol #Tindol #HighPills #promethazine #tramadol #leanUS #Fatec #Phatec #LiquidDrug #Syrup #leansyrup #B5#MagicMushrooms Feel free to ask (line contact link hidden) |
| **Location** | | | | |
|  | Yes | 1322 (34.5) | ขยับราคาใหม่มีกลุ่มเครดิตเช็คได้ 1ชิ้น =1400 2ชิ้น =2200 5ชิ้น ฟรี 1ชิ้นกทม.ปทุมส่งด่วน1ชมตจว.1-2วัน#ไฮ #นัดไฮ #สายคลอง #ไฮปทุม #สายไฮ#ไฮฉีด #ไฮกทม #นัดเย็ดรังสิต #นัดเย็ดกทม #ไฮแล้วเงี่ยน #สายดีด #น้ำแข็ง #ยาไอซ์ #โกงไฮ #ไฮนัว #พิษณุโลกนัดเย็ด #รังสิตนัดเย็ด | Updated pricing: Credit group available for verification: 1 unit=1400 THB. 2 units=2200 THB Buy 5, get 1 free; Bangkok/Pathum Thani: 1-hour express delivery Upcountry: 1-2 days delivery. #High #MeetForHigh #CanalGang #PathumHigh #HighInjection #BangkokHigh #SexMeetRangsit #SexMeetBKK #HighAndHorny #Tweakers #Crystal #Ice #ScamHigh #HighNow #PhitsanulokSexMeet #RangsitSexMeet |
| **Discount** | | | | |
|  | Yes | 1055 (27.5) | ปลีกยูนิแลค 2400 3ลังขึ้น 2350 ฟาเทค 1500 3ลังขึ้น 1450ฝาแดง 3300 3ลังขึ้น 3250เทา 2850 3ลังขึ้น 2800แคปชมพู 2200 3กระปุกขึ้น 2150เพนดอน,ทรามาดอล 1650 ส่ง 5กล่องเพนดอน,ทรามาดอล1600แคป+ยาเป็นชุดก็ขายนะคะสมุทรปราการรับที่ร้านหรือจะเรียกรถให้ไปส่งได้ค่ะ #ทรามาดอล #ยูนิแลค | Retail price for Unilac: 2400 THB 3 boxes or more: 2350 THB Fatec: 1500 THB 3 boxes or more: 1450 THB “Red cap”: 3300 THB 3 boxes or more: 3250 “Gray”: 2850 THB; 3 boxes or more: 2800 THB “Pink capsules”: 2200 THB 3 bottles or more: 2150 THB Pendon, Tramadol: 1650 THB For 5 boxes: 1600 THB; Capsules+pills also available as combo sets. Available for pickup in Samut Prakan or delivery by car;#Tramadol #Unilac |
| **Credibility** | | | | |
|  | Yes | 1284 (33.5) | ยานอนหลับ มีพร้อมส่ง ยานอนหลับอย่างดี เขียวเหลือง เม็ดเมายาสลบเม็ดชนิดรุนแรง การันตีผล หลับสนิท ไม่รุ้สึกตัวยาแท้ ส่งจิง 100% สนใจแอดไลนกลุ่มมีเครดิต จบงานโทร 09699xxxx Line: mr.pxxxxy psclinic ยาสลบแบบเม็ด | High-quality sleeping pills available for immediate delivery. Green-yellow pills, sedative tablets, strong anesthetic-type pills. Guaranteed effect—deep sleep, complete loss of consciousness. Authentic products, 100% real delivery. Interested buyers: add via LINE, verified group with credit references. Contact to complete the deal: 09699xxxx LINE: mr.pxxxxy (psclinic) Tablet-form anesthetics |
| **Fast delivery** | | | | |
|  | Yes | 1216 (31.7) | เครดิตร้านค่ะส่งจริง ส่งไว ส่งของทุกวัน... พร้อมส่งทุกบ้าน ไม่ดองยอดสอบถามเพิ่มเติม Line: @71xxxxptp https://lin.ee/xxxxhdw~#ยาน้ำ #เขียวเหลือง #ทามาดอล #ฟาเทค #ซีซ่า #บี5 #ราคาส่ง #เม็ดเมา #เขียวเหลืองราคาส่ง #ยาแก้ไอ #โปรเม #ฝาแดง #ยาน้ำแก้ไอ #ซีซ่า #เพนดอล | Verified seller. Fast and reliable delivery. We ship every day...ready to deliver to all homes. No order delays. For inquiries, contact via LINE: @71xxxxptp (https://lin.ee/xxxxhdw) #LiquidDrug #GreenYellow #Tramadol #Fatec #Ciza #B5 #WholesalePrice #HighPills #GreenYellowWholesale #CoughMedicine #Prome #RedCap #CoughSyrup #Ciza #Pendol |
| **Direct contact** | | | | |
|  | Yes | 2848 (74.3) | อะโซแลน ตัวยาอันปราโซแลมสายชิวมีเก็บปลายทาง+50ไม่ต้องโอนhttp://line.xxxxxxPPAP64 #พรีนา #Prenarpil #โพวา #สายขาว #เม็ดเมา #ยานอนหลับ #โรเช่ #เช่ไทย #เช่นอก #เช่สเปน #คอนดัก #แล่ม #ซาแน้ก #เขียวเหลือง #แวเลี่ยม #ยาแก้ซึมเศร้า #บี5 #B5 #Rivotril | Azolam (active ingredient: alprazolam)—chill type. Cash on delivery available (+50 THB), no advance payment required. LINE: http://line.xxxxxxPPAP64; #Prena #Prenarpil #Pova #WhitePillsGang #HighPills #SleepingPills #Roche #ThaiRoche #ImportedRoche #Conduct #Lam #Xanax #GreenYellow #Valium #Antidepressants #B5 #Rivotril |
| **Quality** | | | | |
|  | Yes | 926 (24.2) | #ยาปลุกอารมณ์Cantharis D6เกรดพรีเมี่ยมพร้อมส่งการันตีคุณภาพ เก็บเงินปลายทางสายตรง 097-131-xxxx มาร์ชครับ | Cantharis D6—premium-grade aphrodisiac, ready for delivery. Quality guaranteed. Cash on delivery. Direct line: 097-131-xxxx (Marsh) |
| **Method of delivery** | | | | |
|  | Postal only | 32 (0.8) | ของแทร่ไม่จกตา มีเลขพัสดุให้ทุกออเดอร์#โซแล่ม #เม็ดเมา #เม็ดม่วง #โรเช่#พรีนา #คอนดรัก #ทินดอล #โคเดอีน#ฝาแดง #ยานอนหลับ #ยาเมา #ยาแก้ไอ#ยาปลุกเซ็กส์ #โปรเมทาซีน #โรฮิปนอล#ซาแน็ก #ทรามาดอล #โปรโคดีน#ยาแพนิค #เขียวเหลือง #ยาเสียสาว#อัลปร้าโซแล่ม #โปรโคดิ้ว #ยาซึมเศร้า | Our products are real—no scams. Tracking number for every order. #Zolam #HighPills #PurplePills #Roche #Prena #Condrak #Tindol #Codeine #RedCap #SleepingPills #Intoxicants #CoughMedicine #SexDrugs #Promethazine #Rohypnol #Xanax #Tramadol #Procodeine #PanicMeds #GreenYellow #DateRapeDrug #Alprazolam #Procodew #Antidepressants |
|  | Face-to-face only | 51 (1.3) | นัดรับรังสิต ยากนอกเข้ามาคุยกันครัย#พรีนา #Prenarpil #โพวา #สายขาว #เม็ดเมา #ยานอนหลับ #โรเช่ #เช่ไทย #เช่นอก #เช่สเปน #คอนดัก #แล่ม #ซาแน้ก #เขียวเหลือง #แวเลี่ยม #ยาแก้ซึมเศร้า #บี5 #B5 #Rivotril #โคเดอีน #นัดเย็ด #นักเย็ดรังสิต | Meet at Rangsit. If you are outside, come and talk to us. #Prena #Prenarpil #Pova #Whiteline #Horny pills #Sleeping pills #Roche #Thai Che #Che Ok #Che Spain #Conduct #Laem #Xanax #Green and yellow #Valium #Antidepressants #B5 #B5 #Rivotril #Codeine #Meet up for sex #Rangsit sex workers |
|  | Courier | 258 (6.7) | สั่งเยอะสั่งน้อย เท่าไหร่ก็ส่งนะคะ แป๊ะงานสิงโตแท้จีละ 900 ครึ่งจี 500 น. ไม่รวมถุง แถมให้แน่นๆทุกออเดอร์ กทม-ปริมณฑลส่งด่วนลาล่ามูฟไม่เกิน 2ชม. จบงาน ตจว. ส่งพัสดุแฟลช สนใจสั่งซื้อแอดไลน์@𝟒𝟗###𝐞𝐨𝐡#แป๊ะโรย #แป๊ะ #เฮโรอีน #ไก่ขาว #ผงขาว #ตัวเล็ก #น้ำแข็ง #ไฮคลู | Order a lot or a little, we will deliver as much as you want. Real lion head, 900 baht per G, half G 500 baht, weight excluding bag, plus extra for every order. Bangkok and surrounding areas, express delivery by Lalamove, no more than 2 hours, up-country, Flash parcel delivery. Interested in ordering, add Line @ 𝟒𝟗###𝐞𝐨𝐡 #Royal Lion Head #Lion Head #Heroin #White Chicken #White Powder #Small #Ice #Hi-Cool |
|  | Multiple | 1596 (41.6) | แคปเขียวเหลือง ฟาเทคอุบลชนมือ>> มีแกรป,พัสดุ <<แคป1/ฟาเทค 1 ชุดละ 13× ฿#โปรเมทาซีน #เขียวเหลือง #ฟาเทค #urmineflwr #ยาน้ำ #เม็ดเมา #ทรามาดอล #เพนดอล #อุบลชนมือ#เขียวเหลือง #อุบล #อุบลราชธานี #อุบลชนมือ #ทรามาดอล | Green-yellow capsules and Fatec, Ubon in-person deals. Available via Grab or postal delivery. 1 Green-yellow+1 Fatec capsule set: 13× THB. #Promethazine #GreenYellow #Fatec #urmineflwr #LiquidDrug #HighPills #Tramadol #Pendol #UbonInPerson #Ubon #UbonRatchathani #Tramadol |
| **Type of substances** | | | | |
|  | Opioids | 2807 (73.3) | ยอดวันที่16/04ชนมือชนมือ เพชรเกษม กทม. ส่ง+100ไม่เก็บเงินปลายทางกลัวทางร้านโกงยังไม่ต้องทักมาซื้อสอบถ มีทุกอย่างติดต่อไลน์https://line.me/ti/p/xxxxxF-DIV#แป๊ะ #ผงขาว #ไก่ #ไข่ขาว #เฮโรอีน #ตัวใหญ่ #เคตามีน | Order from April 16—in-person handoff (cash-on-hand only)Phetkasem, Bangkok—delivery available (+100 THB). No cash-on-delivery. If you fear the shop is a scam, please don’t message to buy. We’ve got everything. Contact LINE: (link). #Pae #WhitePowder #Chicken #EggWhite #Heroin #BigStuff #Ketamine |
|  | Antihistamines | 2394 (62.5) | พร้อมส่งแล้ว!!!!! มีปลายทาง มีกลุ่มเครดิตLine: @067ts###q#โปรเมทาซีน #ฟาเทค #ฝาแดง #ฝาเทา #ฝาทอง #ใบกระท่อม #เขียวเหลือง #ลีนนอก #ยาแก้ไอ #เม็ดเมา #ยานอนหลับ #กัญชา #ทรามาดอล #เพนดอล #ยาน้ำ #ทินดอล #HK #โปรลีน #อาซาคอก | Ready to ship now! Cash on delivery available. Credit group accessible.LINE: @067ts###q. #Promethazine #Fatec #RedCap #GrayCap #GoldCap #KratomLeaf #GreenYellow #ImportedLean #CoughMedicine #HighPills #SleepingPills #Cannabis #Tramadol #Pendol #LiquidDrug #Tindol #HK #ProLean #Asacoc |
|  | Benzodiazepines | 2009 (52.4) | โซแลม1.0-0.5-0.25เม็ดม่วงสายแมวมีเก็บปลายทาง+50ไม่ต้องโอนhttp://line.mexxxx/~PPAP64#พรีนา #Prenarpil #โพวา #สายขาว #เม็ดเมา #ยานอนหลับ #โรเช่ #เช่ไทย #เช่นอก #เช่สเปน #คอนดัก #แล่ม #ซาแน้ก #เขียวเหลือง #แวเลี่ยม #ยาแก้ซึมเศร้า #บี5 #B5 #Rivotril #โคเดอีน | Zolam 1.0/0.5/0.25 mg—purple pills for chill users. Cash on delivery available (+50 THB), no prepayment required (Line link). #Prena #Prenarpil #Pova #WhitePillGang #HighPills #SleepingPills #Roche #ThaiRoche #ImportedRoche #Conduct #Lam #Xanax #GreenYellow #Valium #Antidepressant #B5 #Rivotril #Codeine |
|  | Amphetamine-type substances | 756 (19.7) | ส่งด่วนสอบถามได้ครับ #ฉีด #ไฮ #ไฮกรุงเทพ #ไอซ์ #ตัวใหญ่ #เม็ดเมา #ยาเมา #ยาเสียว #ฉีดเข้าเส้น #กรุงเทพ #โคเคน #ไฮดีด #ไฮนัว #ผงขาวกทม #เงี่ยนแล้วไฮ #นัดไฮกทม #ไฮแล้วเงี่ยน #ไฮคลู #ไฮเงี่ยน #ไฮแล้วเย็ด #ไฮเชียงใหม่ #ไฮพัทยา #hicool | Express delivery available. Inquire for details.Injection #High #HighInBangkok #Ice #BigStuff #HighPills #Intoxicants #ArousalDrugs #IntravenousInjection #Bangkok #Cocaine #TweakingHigh #NowHigh #BangkokHeroin #HighAndHorny #MeetForHighInBKK #HighThenSex #CoolHigh #HornyHigh #HighInChiangMai #HighInPattaya #hicool |
|  | Ketamine | 546 (14.2) | #หนม#เคตามีน#ไฟว์#แฮปปี้#คอลลาเจน#ตัวใหญ่#ตัวเล็ก#เฮโรอีน#โค้ก#โคเคน งานมาเพิ่มอีกแล้วครับสนใจแอดไลน์มานะครับของพร้อมส่งพี่ๆน้องๆมาลองชิมของของผมแล้วจะติดใจครับถ้าใครจะเอาไปทำ ฝากถึงรายย่อยถ้าตังค์ไม่พอมัดจำก่อนก็ได้รู้เรื่องแล้วหลายรอบ | #Snack #Ketamine #Five #Happy #Collagen #BigStuff #SmallStuff #Heroin #Coke #Cocaine. New stock just arrived. Interested? Add me on LINE. All products ready for delivery. Everyone who tried my goods keeps coming back. For resellers—if you’re short on cash, a deposit is OK. We have done this many times already. |
|  | Sex-performance substances | 543 (14.2) | บลูยาเพิ่มอารมหญิงอารมมาเต็มจัดนักได้ทุกรอบมีเก็บปลายทาง+50ไม่ต้องโอนhttp://line.me/ti/p/~PPAP64#พรีนา #Prenarpil #โพวา #สายขาว #เม็ดเมา #ยานอนหลับ #โรเช่ #เช่ไทย #เช่นอก #เช่สเปน #คอนดัก #แล่ม #ซาแน้ก #เขียวเหลือง #แวเลี่ยม #ยาแก้ซึมเศร้า #บี5 #B5 #Rivotril #โคเดอีน | Blue pill to enhance female arousal—brings full desire, ready for every round. Cash on delivery available (+50 THB), no advance payment required. (LINE contact link). #Prena #Prenarpil #Pova #WhitePillGang #HighPills #SleepingPills #Roche #ThaiRoche #ForeignRoche #Conduct #Lam #Xanax #GreenYellow #Valium #Antidepressants #B5 #Rivotril #Codeine |
|  | MDMA^b^-type substances | 513 (13.4) | Bkk grab#เม็ดเมา #หนมเมา #ยาบ้า #ทรามาดอล #เม็ดเมากรุงเทพ #เม็ดเมารามคำเเหง #ผงขาว #โรฮิป #งานดีด #นัดเย็ดกรุงเทพ #นัดเย็ดรังสิตภิรมย์ #molly | Bkk grab # SleepingPills #drunken snacks #Methamphetamine #Tramadol #drunk pills in Bangkok # SleepingPills in Ramkhamhaeng #white powder #Rohip #exciting work #bangkok sex appointment #rangsit phirom sex appointment #molly |
|  | Cannabis | 426 (11.1) | Check Link in bio! tag; #ดอล #ทามาดอล #เขียวเหลือง #โปรเมทาซีน#ลีน #เม็ดเมา #ดอลเชียงราย #กัญชาพะเยา #ลีนเชียงคำ #ลีนเชียงราย #ลีนพะเยา #ฟาเทค#ดอลพะเยา #กัญชา #แป๊ะ #นัดเย็ดเชียงราย #ยานอนหลับ #ฟาเทค #ลีนเชียงคำ #กัญชาเชียงคำ #ลีนนอก | Check link in bio! #Dol #GreenYellow #Promethazine #Lean #HighPills#DolChiangRai #CannabisPhayao #LeanChiangKham #LeanChiangRai#LeanPhayao #Fatec #DolPhayao #Cannabis #Pae#HookupInChiangRai #SleepingPills #Fatec #LeanChiangKham#CannabisChiangKham #ImportedLean |
|  | Cocaine | 59 (1.5) | โค้ก พร้อมส่งจากไทยประมาณ100g สนใจทักหรือคลิ๊กลิงค์เพื่อดูสินค้าในเทเลแกรมได้เช่นกันhttp://t.me/maxxxxxanmar1…#ขายแป๊ะ #ลิ้นฟ้า #โรฮิป #MDMA #ฉีดเข้าเส้น #ยาอี #ยาอีดัตช์ #ตัวเล็ก #ตัวใหญ่ #ทรามาดอล #โรฮิปนอล #ลีนนอก #เม็ดเมา #ตัวเล็ก #ยาบ้า #ยาม้า #เฮโรอีน #ขายเค #โค้ก #โคเคน | Coke ready to ship from Thailand, ~100 g available. Interested? Message or click link to view products on Telegram. (Telegram link). # HeroinDealer #BlueTongue #Rohypnol #MDMA #IntravenousInjection#Ecstasy #DutchEcstasy #SmallBatch #BigBatch#Tramadol #Rohypnol #ImportedLean #HighPills #SmallBatch#Methamphetamine #Speed #Heroin #KetaminesForSale #Coke #Cocaine |
|  | Hallucinogens | 44 (1.1) | เห็ดเข้าเเล้วนะครับ พร้อมจำหน่ายตอนนี้ในราคาเบาๆเริ่มจีละ120-300ครับผม:บริการปลายทาง บริการส่งด่วนครับ+120ส่งปกติ+50฿. -ห่างไกล+100฿. #ลีนนอก #เม็ดเมา #ยานอนหลับ #โรเช่ #เขียวเหลือง #ทามาดอล #B5 #สายตี้ #สายยานำ้ #สายตึ้ม #ฟาเทค #ดอลF100 | Mushrooms have arrived—now available at affordable prices!Starting from 1 g at 120-300 THB. Cash on delivery service available. Express delivery: +120 THB Standard delivery: +50 THB Remote area: +100 THB. #ImportedLean #HighPills #SleepingPills #Roche #GreenYellow #Tramadol #B5 #PartyCrew #LiquidDrugUsers #Clubbers #Fatec #DolF100 |

^a^All advertisement examples were translated by NM, who is a Thai native speaker.

^b^MDMA: 3-methoxy-4,5-methylenedioxyamphetamine.

This is a Multimedia Appendix to a full manuscript published in the JMIR Infodemiology journal. For full copyright and citation information see http://dx.doi.org/10.2196/71703.
